# Severe acute respiratory syndrome coronavirus 2-reactive salivary antibody detection in South Carolina emergency healthcare workers, September 2019–March 2020

**DOI:** 10.1017/S0950268824000967

**Published:** 2024-09-25

**Authors:** Haley C. Meltzer, Jane L. Goodwin, Lauren A. Fowler, Thomas W. Britt, Ronald G. Pirrallo, Jennifer T. Grier

**Affiliations:** 1 Department of Biomedical Sciences, University of South Carolina School of Medicine Greenville, Greenville, SC, USA; 2 Department of Physiology and Pharmacology, Wake Forest School of Medicine, Charlotte, NC, USA; 3 Department of Psychology, Clemson University, Clemson, SC, USA; 4 Department of Emergency Medicine, University of South Carolina School of Medicine Greenville, Greenville, SC, USA

**Keywords:** antibody surveillance, ELISA, emergency healthcare workers, saliva, SARS-CoV-2

## Abstract

On 19 January 2020, the first case of severe acute respiratory syndrome coronavirus 2 (SARS-CoV-2) infection was identified in the United States, with the first cases in South Carolina confirmed on 06 March 2020. Due to initial limited testing capabilities and potential for asymptomatic transmission, it is possible that SARS-CoV-2 may have been present earlier than previously thought, while the immune status of at-risk populations was unknown. Saliva from 55 South Carolina emergency healthcare workers (EHCWs) was collected from September 2019 to March 2020, pre- and post-healthcare shifts, and stored frozen. To determine the presence of SARS-CoV-2-reactive antibodies, saliva-acquired post-shift was analysed by enzyme-linked immunosorbent assay (ELISA) with a repeat of positive or inconclusive results and follow-up testing of pre-shift samples. Two participants were positive for SARS-CoV-2 N/S1-reactive IgG, confirmed by follow-up testing, with S1 receptor binding domain (RBD)-specific IgG present in one individual. Positive samples were collected from medical students working in emergency medical services (EMSs) in October or November 2019. The presence of detectable anti-SARS-CoV-2 antibodies in 2019 suggests that immune responses to the virus existed in South Carolina, and the United States, in a small percentage of EHCWs prior to the earliest documented coronavirus disease 2019 (COVID-19) cases. These findings suggest the feasibility of saliva as a noninvasive tool for surveillance of emerging outbreaks, and EHCWs represent a high-risk population that should be the focus of infectious disease surveillance.

## Introduction

Since its arrival to the United States, severe acute respiratory syndrome coronavirus 2 (SARS-CoV-2), the causative agent of the novel coronavirus disease 2019 (COVID-19) pandemic, has challenged the world’s infectious disease surveillance systems. Its high asymptomatic transmissibility contributed to the rapid international spread that highlighted the extent to which healthcare systems were ill-equipped to manage a novel infectious agent [1, 2]. Efficient, coordinated surveillance of viruses with pandemic potential is an integral aspect of public health and preparedness for outbreaks [3].

On 19 January 2020, the first confirmed case of COVID-19 in the United States was identified in Snohomish County, Washington [4], with the first known case in South Carolina identified on 06 March 2020 [5]. However, initial infection reports are likely incomplete due to barriers to accurate viral surveillance. Testing availability in the early months of 2020 was limited due to supply and personnel shortages, as well as an overall lack of knowledge about SARS-CoV-2 [1, 6, 7]. Concurrently, individuals with asymptomatic or mild symptomatic cases may not have sought testing, contributing to further infection underreporting [8–10]. More recent data suggest that the true date of SARS-CoV-2’s arrival to the United States was earlier than January 2020. The Retrospective Methodology to Estimate Daily Infections from Deaths algorithm, derived from global seroprevalence and death data, suggests that SARS-CoV-2 may have been in the United States in December 2019 and in South Carolina by 30 January 2020 [6]. Retrospective serological analysis of archived blood samples found the presence of SARS-CoV-2-reactive antibodies in every state tested, suggesting that the virus may have been spreading in the United States as early as December 2019 [8].

To accurately report the burden of infection in a community, it is important to understand when the pathogen may have been introduced and the prevalence of antibody responses against it. Serological studies have proven effective at revealing the presence of antiviral antibodies that may represent possible infections early during a pandemic [8, 11]. The presence of anti-SARS-CoV-2 antibodies in an at-risk population suggests prior coronavirus antigen exposure, potentially from SARS-CoV-2 infection or an infection with a related pathogen capable of inducing cross-reactive antibodies [12].

With documented asymptomatic SARS-CoV-2 infections [13], testing of only ill patients during the early stages of the pandemic may have contributed to an underestimation of the virus’s prevalence. To obtain a complete picture of SARS-CoV-2’s viral burden, it is important to test individuals with and without symptoms. Serological testing of over 7000 blood specimens detected anti-SARS-CoV-2 antibodies in 1–2% of US donors, with variations in dates and locations of sample collection [8]. However, this study did not include locations in the southern United States, which became an epicentre for infection burden as the pandemic progressed. In fact, as of January 2021, the Greenville, South Carolina metro area had the highest number of cases per 100000 (762) among towns with a population between 250000 and 1000000 [14].

The potential for asymptomatic SARS-CoV-2 transmission also raises concerns about the role that healthcare workers might play in the spread of the virus. Healthcare providers, particularly those with direct patient contact, demonstrated an increased risk of COVID-19 infection compared to the general population [15], yet less than 15% of all reported COVID-19 cases in the Centers for Disease Control and Prevention (CDC) surveillance system included data on whether the patient was a healthcare worker [16]. Consequently, COVID-19 exposures, infections, and transmission among healthcare personnel may be largely underestimated.

Selecting the proper surveillance location and population is integral to the effectiveness of early detection and identification of a disease. Emergency healthcare workers (EHCWs) represent one of the first lines of interaction between the community and the healthcare system. Thus, early and focused sampling of emergency department (ED) clinicians and emergency medical service (EMS) prehospital providers could contribute to a better understanding of future infectious diseases, particularly those capable of asymptomatic, person-to-person transmission. Furthermore, given the interaction between EHCWs and the public with potentially undiagnosed infections, an understanding of virus-reactive antibody responses in this population can provide important insights into viral prevalence or potential pre-existing immunity.

Early in the pandemic, nasopharyngeal and oropharyngeal swabs were established as the gold standard for SARS-CoV-2 screening. More recently, viral detection in saliva has also demonstrated efficiency and reliability [17, 18]. Benefits of testing salivary samples for infectious diseases and markers of immunity include greater cost efficiency and lower transmission risk to healthcare workers during sample collection [19]. However, accurate and reliable detection of salivary biomarkers may be dependent on collection and storage conditions, such as the use of nucleic acid preservation media for the detection of viral ribonucleic acid (RNA) [20]. Under the right conditions, saliva is conducive to long-term sample storage, making it an effective tool for retrospective testing.

To determine whether antibody recognition of SARS-CoV-2 viral components was present in EHCWs in Greenville County, South Carolina, prior to the first documented cases of COVID-19 and assess the potential of salivary sample testing in immune surveillance, salivary samples collected between September 2019 and March 2020 were retrospectively tested for the presence of SARS-CoV-2-reactive antibodies.

## Methods

### Setting and participant population

Greenville County is located in northwest South Carolina and consists of mixed rural and suburban communities, with the largest city being the City of Greenville. Prisma Health Greenville Memorial Hospital’s ED is the only American College of Surgeons (ACS)-verified Adult Level 1 and Paediatric Level 2 Trauma Centre in the Upstate, providing emergency care for over 106000 patients annually.

The Greenville County Emergency Medical Services (GCEMS) system serves this area with 23 advanced life support transport units, and in 2018, they responded to over 85000 calls. Greenville is also the home of the University of South Carolina School of Medicine Greenville (USCSOMG). The USCSOMG medical student curriculum requires all students to complete Emergency Medical Technician (EMT) training, and students are integrated into the GCEMS patient care team during their pre-clerkship years. Study participants included 9 Greenville Memorial Hospital ED physicians, 7 GCEMS providers, and 39 USCSOMG medical students working as EMTs (Table 1).

**Table 1.** Demographic information of participant population

	ED Physicians	GCEMS Providers	USCSOMG Students
Number of Participants	9	7	39
**SARS–CoV–2 N/S1 IgG**^**+**^	0	0	2
**SARS–CoV–2 S1 IgG**^**+**^	0	0	1
Gender (% (#))			
Male	33.3% (3)	42.9% (3)	48.7% (19)
Female	66.6% (6)	42.9% (3)	51.3% (20)
Other	0.0% (0)	14.3% (1)	0.0% (0)
Average Age	N.D.	33.75	24.05
**SARS–CoV–2 N/S1 IgG**^**+**^	n.a.	n.a.	25
**SARS–CoV–2 S1 IgG**^**+**^	n.a.	n.a.	25
Shift Duration (hr)	8	12	12

Abbreviations: n.a., not applicable; N.D., not determined.

### Human studies’ approval

All salivary samples were collected during two independent studies approved by the Prisma Health Institutional Review Board, in which patient consent allowed for samples to be analysed for supplementary research. All samples and data were gathered and stored in a de-identified manner.

### Sample collection and storage

A total of 160 salivary samples were obtained from 55 EHCWs in Greenville, South Carolina, between September 2019 and March 2020. Using the passive drool method, saliva was collected immediately before and after participants’ shifts, which occurred at variable times of the day and varying days of the week. One to two millilitres of saliva was collected and stored in a 2–4°C refrigeration unit immediately before being moved to a -80°C freezer within 72 hours. Initial assays of anti-SARS-CoV-2 N/S1 Receptor Binding Domain (RBD) IgG occurred between January and June 2022, following approximately 2 years of storage; subsequent assays of anti-SARS-CoV-2 S1 RBD IgG and IgM were conducted in May 2024, following approximately 4 years of storage.

### Prevention of contamination

All reactions were prepared in a 1300 Series Class II, Type A2 Biological Safety Cabinet. Prior to use, the hood space was ultraviolet (UV)-treated. Hood surfaces were then sterilized with a 10% bleach solution followed by 70% ethanol, as were all pipettes and supplies. Only sterile tubes and filtered tips were used. Gloves were changed frequently, as well as after coming in contact with samples. Personal protective equipment was worn throughout all experiments in accordance with Biosafety Level (BSL)-2 safety protocols.

### ELISA

The presence of IgG or IgM against the SARS-CoV-2 N protein and/or the RBD of the S1 protein was assayed via *in vitro*, indirect enzyme-linked immunosorbent assay (ELISA). Frozen salivary samples were thawed at 4°C for 24–48 hours, vortexed until homogenous, and then diluted based on the manufacturer’s recommendations or experimental determinations (Supplementary Figure S1). Diluted samples were added to ELISA plates pre-coated with SARS-CoV-2 protein targets (RayBiotech; Table 2), and testing was performed according to the manufacturer’s specifications. A positive control salivary sample for SARS-CoV-2 antibodies, obtained in February 2022 from a healthy 23-year-old woman with a history of polymerase chain reaction (PCR)-confirmed prior SARS-CoV-2 infection and vaccination, was subjected to similar freeze–thaw and dilution processes as experimental samples. Initial screening of all post-shift samples was performed via assay for IgG against a combination of the SARS-CoV-2 N and S1 RBD antigens. Bound human IgG or IgM antibodies were quantified by optical density (OD) at 450 nm using a microplate reader. Designated control OD signals were subtracted from SARS-CoV-2 signals to correct for background interference. A minimum of technical duplicates were performed for all samples tested.

**Table 2.** RayBiotech SARS-CoV-2 Human Immunoglobulin ELISA Kit specifications

Manufacturer Ref. #	Ig Isotype Detected	Ig Antigen	Marketed Sample Type	Final Dilution Factor	OD Background	Threshold Value
IEQ–CoVSN–IgG–S	IgG	N + S1 RBD	Saliva	1:10	Albumin	> 0.05 U/mL
IEQ–CoVS1RBD–IgG	IgG	S1 RBD	Serum	1:10	Avg. Zero PC	> 15 U/mL
IEQ–CoVS1RBD–IgM	IgM	S1 RBD	Serum	1:10	Albumin	> 179 U/mL

Abbreviations: Ig, immunoglobulin; PC, positive control.

As the reagents used for the detection of SARS-CoV-2 S1 RBD-specific antibody responses were marketed for use with serum, dilutions of the positive control saliva sample, from undiluted to 1:100, were employed to assess the feasibility of detecting salivary antibody binding to SARS-CoV-2 S1 RBD alone and identify a linear range for sample dilution (Supplementary Figure S1A). Discrimination of positive and negative OD readings for SARS-CoV-2 S1 RBD antibody binding was confirmed for IgG via the evaluation of positive control saliva and a SARS-CoV-2 N/S1-negative saliva sample at sample dilutions of 1:5 and 1:10 (Supplementary Figure S1B **)**. A sample dilution of 1:10 was selected for experimental analysis and employed for SARS-CoV-2 S1 RBD IgG and IgM ELISA of N/S1-positive samples or all post-shift samples, respectively.

For each experimental assay, a calibration curve was calculated and plotted with SARS-CoV-2 protein antibody concentration (unit/mL) on the x-axis and OD signal on the y-axis. For background-corrected samples, calculated concentration values greater than the assay threshold value (Table 2) were defined as a positive result and concentrations less than the threshold value were defined as a negative result. Threshold values were determined by the manufacturer via testing of known positive or negative individuals following established protocols [21]. For positive or inconclusive results, testing was repeated on the post-shift sample, and the pre-shift saliva was evaluated across two independent repeats, with at least one measurement of pre- and post-shift occurring on the same assay plate to minimize batch effects. At the time of use, all ELISA kits were labelled for research use only and not Food and Drug Administration (FDA)-approved for diagnostics.

### Statistical analysis

Analyses of ELISA data, including calculation of calibration curves and anti-SARS-CoV-2 N and S1 RBD antibody concentrations, were performed according to the manufacturer’s specifications. GraphPad Prism 9.4.1 and Microsoft Excel were used for figure generation and statistical analyses.

## Results

### Participant characteristics

All participants felt well at the time of sample collection and did not report any respiratory or gastrointestinal symptoms. Participant demographics varied (Table 1). Additional demographic information, such as race and ethnicity, was not collected to preserve confidentiality.

Two USCSOMG medical student EMTs were found to produce detectable levels of SARS-CoV-2 N/S1-reactive IgG, with the presence of antibodies confirmed in two samples obtained from the same individual 12 hours apart. This constitutes a 5.13% positivity rate among the medical student population and a 3.64% positivity rate among an overall participant. Both participants with SARS-CoV-2 N/S1-reactive IgG antibodies were 25-year-old female medical students.

### Detection of SARS-CoV-2-reactive salivary antibodies

**Figure 1.**
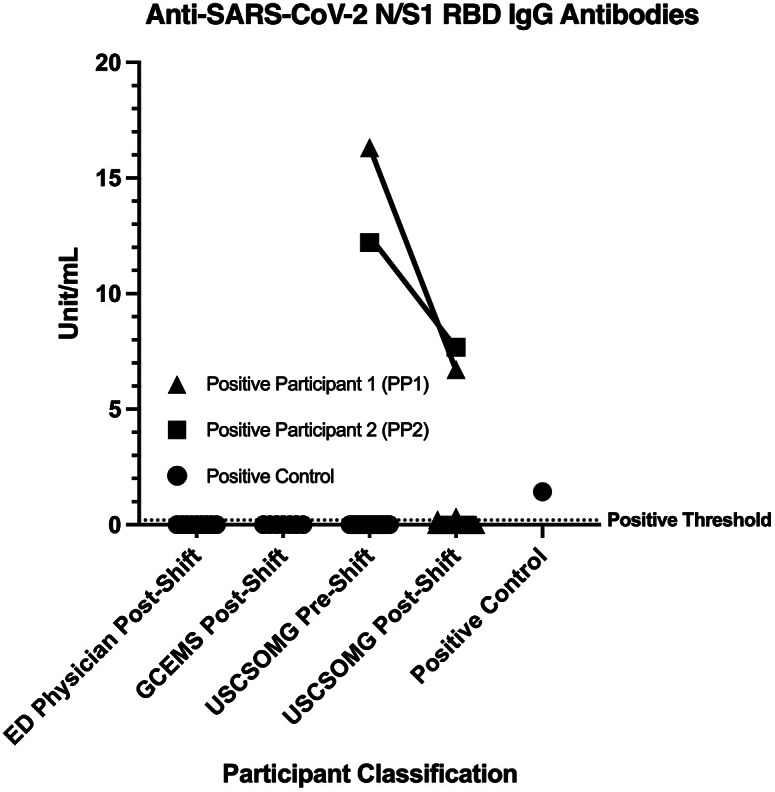
Detection of SARS-CoV-2 N- and S1 RBD-reactive salivary IgG. Median values are shown. Only samples from PP1 and PP2 were found to have pre- and post-shift median values above the positive threshold (0.05 unit/mL).

All post-shift salivary samples collected from GCEMS personnel and ED physicians were negative for the presence of anti-SARS-CoV-2 IgG antibodies. A total of five post-shift samples from medical students resulted in at least one replicate well testing above the positive threshold for N/S1-reactive IgG and were subjected to follow-up testing. Repeat testing identified two participants positive for SARS-CoV-2 N/S1-reactive IgG in both pre- and post-shift salivary samples. Positive Participant 1 (PP1) provided salivary samples on 30 October 2019, and Positive Participant 2 (PP2) provided samples on 23 November 2019 (Figure 1). For both positive participants, median values of SARS-CoV-2 N/S1 RBD-reactive IgG in post-shift samples appeared to be lower than median values of pre-shift samples. Post-shift salivary samples from all participants tested negative for S1 RBD-reactive IgM (data not shown).

**Figure 2.**
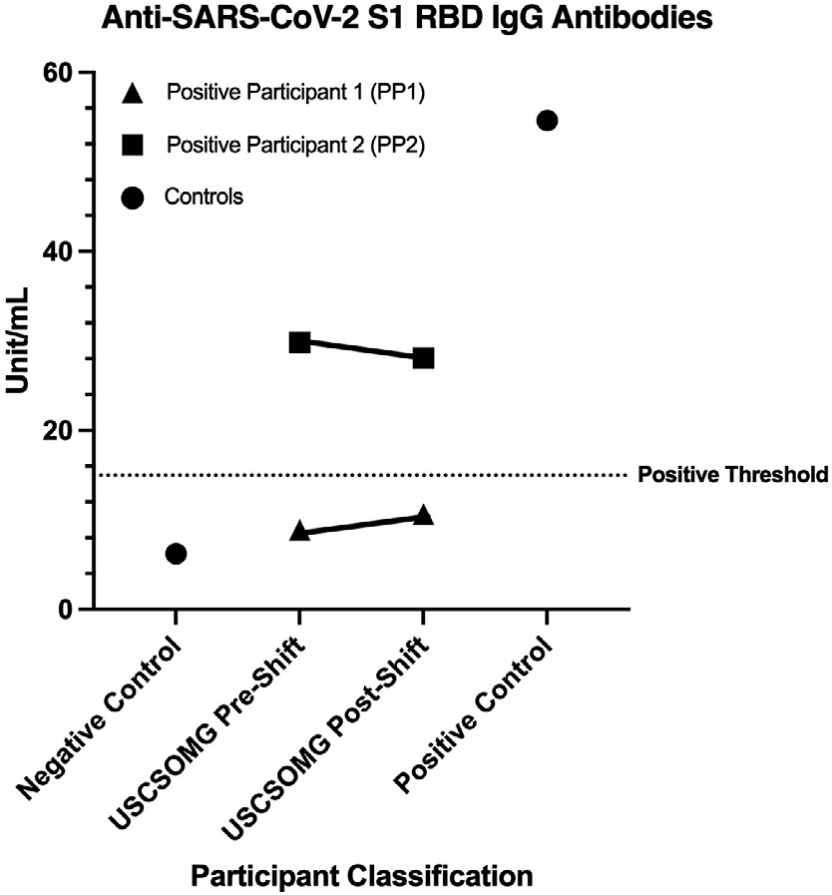
Detection of SARS-CoV-2 S1 RBD-reactive salivary IgG. Median values are shown. Only samples from PP2 were found to have pre- and post-shift median values above the positive threshold (15 unit/mL).

Further analysis of samples from positive participants identified the presence of SARS-CoV-2 S1 RBD-specific IgG in saliva provided by PP2. This was confirmed by repeat testing of both pre- and post-shift samples. PP1, while positive for N/S1-reactive IgG, was negative for IgG specific to S1 RBD (Figure 2).

## Discussion

In this secondary analysis of saliva samples collected from EHCWs, we identified antibodies binding SARS-CoV-2 N and S1 RBD proteins in two individuals during the fall of 2019. Positive results were confirmed in two samples from the same individuals collected 12 hours apart. Repeat testing ruled out potential false positives. While the N protein of coronaviruses maintains sequence similarity between viral family members, the S1 RBD displays significant variability within the virus family and even across SARS-CoV-2 isolates [22,23]. Further investigation of SARS-CoV-2-reactive antibodies in positive samples identified the presence of salivary IgG capable of specifically binding the S1 RBD of SARS-CoV-2 in one individual. The presence of SARS-CoV-2 S1 RBD-binding IgG in this participant suggests that the individual encountered SARS-CoV-2 S1 protein or a highly similar antigen from a related coronavirus prior to providing a salivary sample. This is the first study to detect the presence of SARS-CoV-2 N and S1 RBD-reactive salivary IgG prior to locally reported cases of COVID-19.

Serum studies have identified IgG against SARS-CoV-2 S1 in 100% of hospitalized symptomatic patients with COVID-19 by 6 days after hospital admission, and even 36% of patients with asymptomatic infections developed antibodies against the S1 protein [24]. Furthermore, patients with asymptomatic or mild SARS-CoV-2 infections produced significantly higher levels of serum IgG against both the S1 and N viral proteins compared to exposed healthy controls [25]. Together, these studies suggest that the presence of SARS-CoV-2-reactive IgG observed in the current study indicates a potential for a prior coronavirus infection, rather than simply exposure to infected patients during healthcare work. Based on the timing of positive sample collection in October and November 2019, if the SARS-CoV-2 IgG-positive individuals in this study had experienced SARS-CoV-2 infection, those infections would have occurred prior to the earliest documented cases of COVID-19 in the United States. Participants could not be retrospectively queried about possible exposure or disease history; thus, it is not possible to determine whether a SARS-CoV-2 or other coronavirus infection occurred prior to sample collection and, if so, determine whether the infection was a result of travel or community acquisition. As positive participants were asymptomatic at the time of sample collection, the detection of SARS-CoV-2-reactive antibodies in these salivary samples supports the idea that EHCWs and students who provide emergency care could be an optimal population for surveillance of antibody responses to emerging respiratory pathogens among at-risk individuals, independent of disease status or history.

### Utility of SARS-CoV-2-reactive salivary IgG as an immune biomarker

The detection of anti-SARS-CoV-2 antibodies in saliva has been presented as an alternative approach to serum testing for surveillance of antiviral immune biomarkers in a population [26–30]. The use of salivary samples for the detection of antiviral antibodies has been successfully implemented for a variety of viruses, including hepatitis A and human immunodeficiency virus (HIV), and provided the groundwork for salivary SARS-CoV-2 testing [31–34]. Studies focused on antibody responses during and after infection have validated the correlation between serum and salivary antibody levels [26, 27, 35]. While salivary anti-SARS-CoV-2 IgG levels were found to be lower than serum IgG levels and overall SARS-CoV-2 antibodies are known to wane over time, salivary IgG against SARS-CoV-2 remained detectable for at least 8 to 9 months post-infection [30, 36–38]. Advantages of saliva testing include the ease of sample collection and the ability to store whole samples for future analysis, as evidenced by the two- to four-year gap between sample collection and detection of anti-SARS-CoV-2 antibodies in this study, whereas serum samples require isolation from whole blood prior to long-term storage [39]. Additionally, saliva can be collected in a noninvasive manner, reducing exposure risks to healthcare workers relative to the collection of serum samples, which require close contact with a potentially infectious patient.

This study assessed the presence of IgG antibodies that bind either S1 RBD or N protein of SARS-CoV-2 in the saliva of EHCWs who may have been at risk of exposure in the earliest stages of the pandemic. Antibodies against these viral components are routinely detectable in saliva following SARS-CoV-2 infection [28, 30]. While immunoglobulin A (IgA) and immunoglobulin M (IgM) antibody levels in saliva decay shortly after infection, IgG antibodies against SARS-CoV-2 proteins persist and remain detectable for up to 9 months, demonstrating that salivary IgG is a reliable target for detecting antigen-specific antiviral antibody responses [26, 27, 30]. In addition to providing evidence of prior exposure to viral antigens, salivary antibodies could potentially serve as an indication of immunity against SARS-CoV-2. Salivary antibodies may prove to directly contribute to immune protection, and given the strong correlation between salivary and serum antibody levels, SARS-CoV-2 S1-reactive salivary IgG can signify the presence of anti-SARS-CoV-2 S-neutralizing antibodies in the serum as well [29].

Prior exposure to SARS-CoV-2 is not the only way by which anti-SARS-CoV-2 antibodies may arise. IgG reactive against SARS-CoV-2 N and S1 protein has been detected in the serum of uninfected patients with samples collected as early as 2011, suggesting that cross-reactive antibodies may be generated during infection with other human coronaviruses (HCoVs) [40–42]. A greater percentage of young patients (6–16 years) were found to carry serum IgG antibodies against SARS-CoV-2 compared to young adults (17–25 years) in pre-pandemic samples, which correlates with the increased likelihood of HCoV infection and seroconversion during adolescence [40]. Under pandemic conditions, salivary antibodies against either N or S1 RBD protein were rarely detected in individuals with a COVID-negative history, despite high rates of antibody reactivity with antigens from common coronaviruses in the same population [27]. Yet, in this study, two individuals without prior documented COVID-19 infections were found to be positive for SARS-CoV-2 N/S1-reactive IgG based on salivary samples. The 3.64–5.13% rate of SARS-CoV-2-reactive antibodies in this study fell below the previously reported serum detection range of 5.72–43.75% for patients with a COVID-19 negative history over the age of 17 [40]. However, this difference is likely a result of limited participant numbers and the use of alternate samples for antibody detection. Interestingly, SARS-CoV-2-negative healthcare workers with higher levels of pre-existing plasma IgG against HCoV N protein had lower levels of SARS-CoV-2 N-reactive IgG during the COVID-19 pandemic, indicating a potential protective association between prior HCoV infection and risk of SARS-CoV-2 infection [41]. Furthermore, SARS-CoV-2 S-reactive antibodies from pre-pandemic serum samples proved capable of neutralizing infection by SARS-CoV-2 S pseudotypes or native SARS-CoV-2 infection [40]. This supports the conclusion that participants in this study who tested positive for SARS-CoV-2-reactive IgG may potentially have possessed some immunity against SARS-CoV-2 infection, although further testing of both serum and saliva would be needed to assess whether the specific antibodies present in the participant samples could convey protection.

For both PP1 and PP2, antibody levels appeared to decrease over the shift period, which may indicate a variability of antibody levels as a result of participant internal or external factors. Given that circadian rhythms can impact adaptive immunity [43], the time of day for sample collection was considered as a potential impacting factor on salivary IgG detection; however, positive participants worked opposite schedules on their respective dates of sample collection. Thus, the potential factors, if any, driving variability of antibody responses over the course of a workday may stem from the participants themselves or the specific work conditions. Larger studies of pathogen-specific salivary antibody responses among EHCWs may reveal important aspects of healthcare work that impact adaptive immune responses and may further reveal the potential utility of SARS-CoV-2-reactive salivary IgG as an immune biomarker.

The detection of SARS-CoV-2 N/S1-reactive salivary IgG antibodies in two participants in this study, as well as S1-reactive IgG in one of those participants, demonstrates that a small percentage of EHCWs may have had exposure to SARS-CoV-2 or highly similar antigens prior to the first documented local cases of COVID-19. Given that salivary antibody levels are usually lower than those of serum and the waning of antibody responses over an unknown length of time between antigen encounter and sample collection, this study may actually underestimate the exposure history of the tested participants. Nevertheless, evaluating the prevalence of pathogen-reactive antibodies in a population, regardless of whether they arose from prior infection, transient exposure, or cross-reactive immune responses, is an important step towards understanding susceptibility to infection and the potential for viral transmission within a community during emerging disease outbreaks.

### Limitations

A limitation of this study is that we cannot determine the presence of true positives for SARS-CoV-2 via a positive molecular test for the detection of viral components, such as viral RNA via PCR test [8, 44]. The samples with confirmed positive ELISA results for the presence of anti-SARS-CoV-2 antibodies cannot be definitively considered positive for SARS-CoV-2 infection without confirmation by molecular diagnostic testing [8, 44]. Unfortunately, molecular analysis for SARS-CoV-2 RNA in these samples has not proven successful, despite multiple approaches and attempts. One potential reason why RNA testing may have failed is due to sample collection and storage conditions. PCR detection of viral RNA can be inhibited by the presence of blood, mucus, or food particles in saliva [45]; thus, it is advisable that participants avoid eating or drinking in the 30 minutes prior to sample collection. Due to clinical care duties, it is unknown whether this waiting period occurred and samples were not collected with the intention of detecting RNA; thus, they were not stored in a nucleic acid preservation storage medium. Insight into current or recent infections with SARS-CoV-2 could also be attained by the detection of IgM against viral proteins. However, with the transient nature of salivary IgM responses and the low sensitivity of salivary assays for anti-SARS-CoV-2 S1 or S1 RBD IgM [26], the negative results observed here for SARS-CoV-2 S1 RBD-binding IgM in all tested samples cannot be conclusively interpreted as evidence of an absence of infection history.

While the antibody levels of both PP1 and PP2 decreased over the shift period, the overall low number of positive participants does not allow for the evaluation of trends in antibody levels or similar analyses. Additionally, sources of samples were anonymous, and as such, coronavirus infection history or exposure risks could not be assessed for participants testing positive for SARS-CoV-2-reactive antibodies. Although SARS-CoV-2-reactive antibodies were detected in two participants, without viral RNA detection or participant history regarding travel, health, or sick contacts, conclusions cannot be drawn regarding the presence or prevalence of SARS-CoV-2 in the Greenville, South Carolina area, prior to previously documented cases.

## Conclusions

The exact date of SARS-CoV-2’s arrival to the United States may never be known; therefore, understanding the prevalence of antiviral antibody responses in the population against a notable pathogen is essential to inform surveillance measures and public health decisions. Considerations in infectious disease surveillance highlight the significance of EHCWs as a high-risk population for exposure, infection, and potential transmission. Our data offer a unique glimpse into SARS-CoV-2-reactive immune responses in EHCWs during the time prior to reported cases and suggest that EHCWs themselves could be a focus of disease surveillance and future immunity studies. Furthermore, the ability to detect pathogen-reactive antibodies in saliva >4 years after saliva collection and challenges with successfully completing molecular analysis emphasize the importance of proper saliva sample collection and storage for viral antigen and antibody detection. This work establishes the potential for salivary screening programmes in EHCWs to identify pathogen-reactive antibodies that may impact immunity or disease susceptibility in this high-risk population when novel pathogens emerge.

## Supporting information

Meltzer et al. supplementary material Meltzer et al. supplementary material

## Data Availability

To access materials necessary to replicate findings, readers may contact the authors.

## Supplementary material

For supplementary material accompanying this paper visit http://doi.org/10.1017/S0950268824000967. click here to view supplementary material
