# Supplementary figures and images for: Severe acute respiratory syndrome coronavirus 2-reactive salivary antibody detection in South Carolina emergency healthcare workers, September 2019–March 2020
Source: Epidemiol Infect. 2024 Sep 25;152:e102. doi: 10.1017/S0950268824000967 (PMC11427973; doi:10.1017/S0950268824000967)

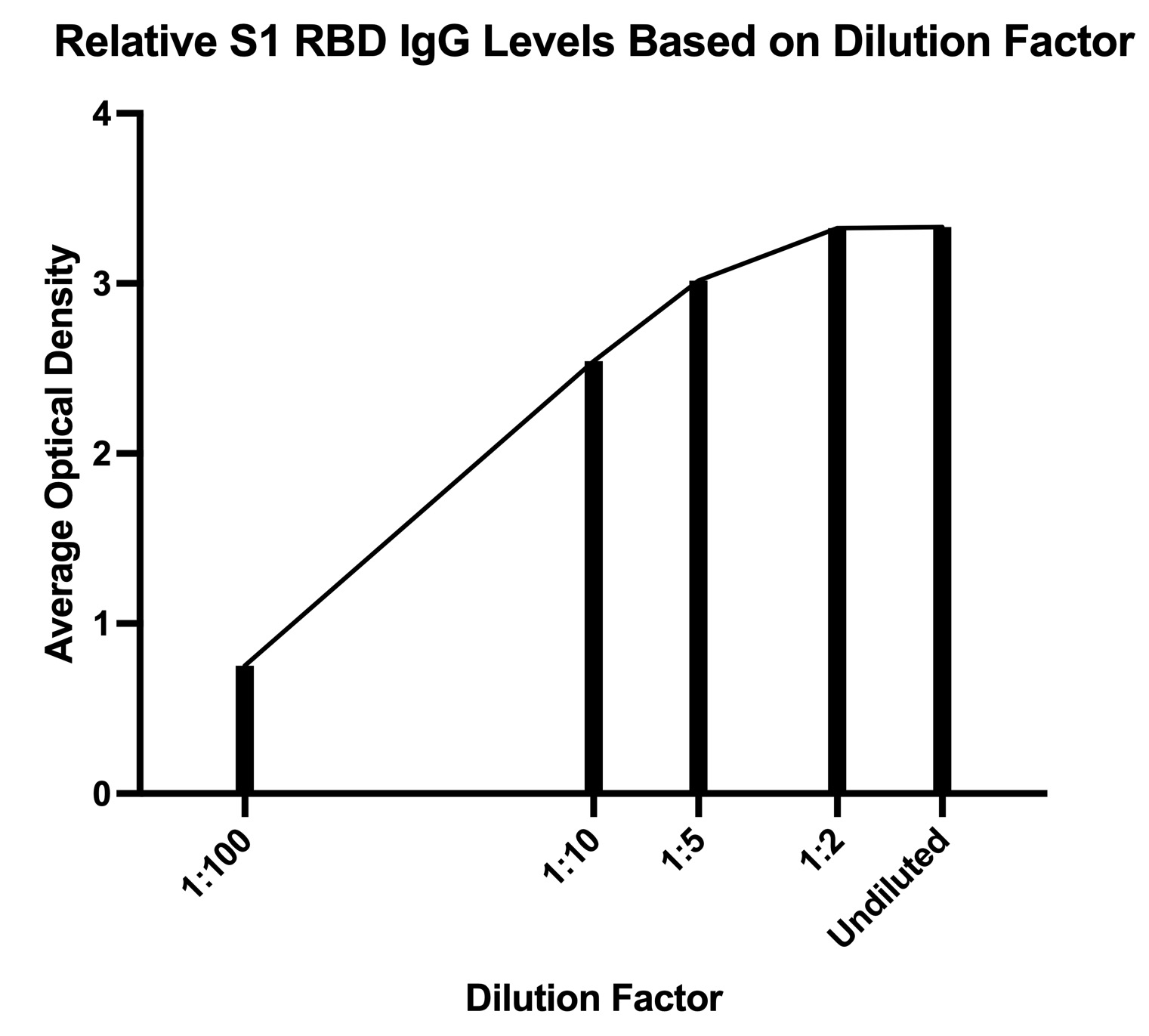

Supplement: Meltzer et al. supplementary material [file S0950268824000967sup001.zip › [Meltzer] SupplementalFig1A.jpg]

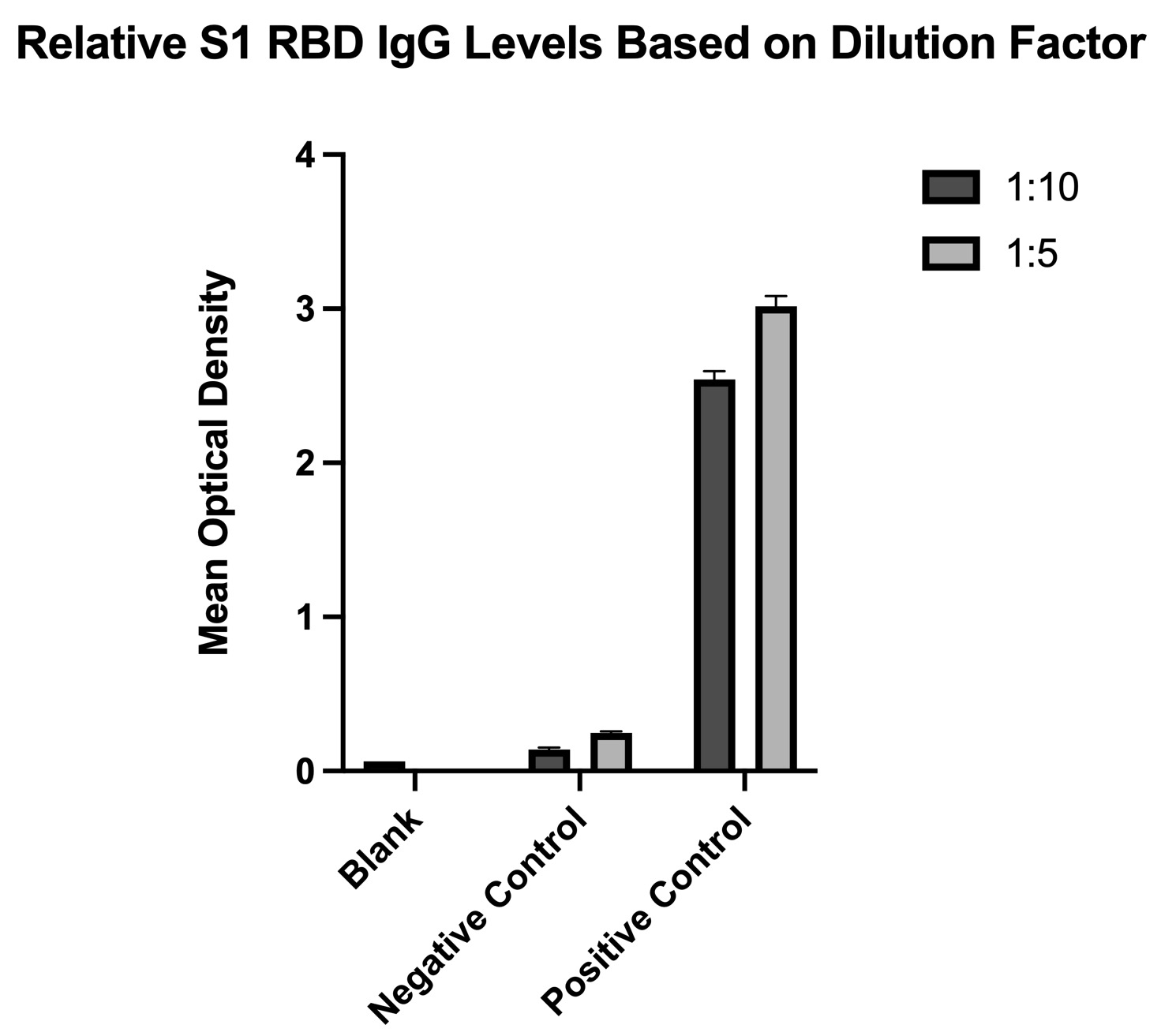

Supplement: Meltzer et al. supplementary material [file S0950268824000967sup001.zip › [Meltzer] SupplementalFig1B.jpg]
